# Acupuncture treatment of diabetic peripheral neuropathy: an overview of systematic reviews based on evidence mapping

**DOI:** 10.3389/fneur.2024.1420510

**Published:** 2024-10-02

**Authors:** Junjie Jiang, Hao Shen, Yi Zhang, Yuanyuan Li, Yuanyuan Jing, Xinyi Chen, Hongli Wu, Yanming Xie, Huan Liu

**Affiliations:** ^1^Institute of Basic Research in Clinical Medicine, China Academy of Chinese Medical Sciences, Beijing, China; ^2^Beijing Tiantan Hospital, Capital Medical University, Beijing, China; ^3^School of Chinese Medicine, Beijing University of Chinese Medicine, Beijing, China

**Keywords:** acupuncture, diabetic peripheral neuropathy, meta-analyses, evidence mapping, AMSTAR-2, PRISMA2020, grade

## Abstract

**Objective:**

The study attempted to evaluate the meta-analyses (MAs) of the acupuncture treatment of diabetic peripheral neuropathy (DPN) to provide a basis for clinical decision-making.

**Methods:**

Eight databases, such as PubMed, Cochrane Library, Embase, Web of Science, CNKI, Wanfang Data, CQVIP, and CBM, were searched from database creation to December 22, 2023. The MAs of DPN treatment using acupuncture or acupuncture combined with conventional Western medicine were included. AMSTAR-2 and PRISMA 2020 helped evaluate the methodological and reporting quality of the included studies. The GRADE methodology helped assess the evidence quality of outcome indicators. Evidence mapping was performed to display evaluation results.

**Results:**

A total of 18 MAs involving 23,240 DPN patients were included. Based on the methodological quality evaluation, four MAs were of “moderate” quality, seven had a quality grade of “low,” and another seven were of “critically low” quality. The evidence quality evaluation showed that among studies of acupuncture vs. conventional Western medicine, four had an evidence quality of “moderate,” 18 had an evidence quality of “low,” and 17 had an evidence quality of “critically low” and that among studies of acupuncture + conventional Western medicine vs. conventional Western medicine, 12 had an evidence quality of “moderate,” 29 had an evidence quality of “low,” and 33 had an evidence quality of “critically low.” Compared with conventional Western medicine, simple acupuncture and acupuncture + conventional Western medicine significantly improved total effective rate (TER) and nerve conduction velocity (NCV).

**Conclusion:**

Acupuncture treatment of DPN significantly improves TER and NCV with proven safety. However, the MAs of the acupuncture treatment of DPN must strictly refer to relevant standards and specifications regarding methodological and reporting quality, along with the design, execution, and reporting of primary randomized controlled trials (RCTs).

## 1 Introduction

Diabetic peripheral neuropathy (DPN) is a leading chronic complication of diabetes. It is defined as the presence of symptoms and/or signs of peripheral nerve dysfunction in diabetes patients after excluding other causes. Its common symptoms include burning pain, electric shock-like or acupuncture-like sensation, numbness, and hyperesthesia, which are often symmetrical. Typical neuropathic pain worsens at night, and its symptoms commonly appear in the feet and the lower limbs and may even involve the hands in certain patients, presenting glove or sock-like distribution (1, 2). DPN is the leading cause of foot ulcers, disability, and amputation. About 50% of type 2 diabetes patients develop DPN within 10 years after initial diagnosis, and at least 20% of type 1 diabetes patients develop DPN within 20 years (3). Moreover, 20–25% of newly diagnosed type 2 diabetes patients may have DPN (4, 5). DPN pathogenesis mainly involves hyperglycemia-induced metabolic disorders, vascular injury, neurotrophic disorders, oxidative stress, inflammatory response, autoimmune injury, and genetic factors (6–9). Acupuncture, by regulating inflammatory response, endoplasmic reticulum stress, and neurological and vascular functions, can significantly alleviate numbness of limbs, pain, sensory disturbance, and other symptoms in DPN patients, with definite efficacy (10).

An overview of systematic reviews (SRs) includes an evidence synthesis method that comprehensively collects SRs/meta-analyses (MAs) in a research field for an overview. It provides users with a summary of evidence and higher-level evidence (11) and has been widely utilized in medicine and health (12, 13). Scholars have conducted overviews of SRs involving the acupuncture treatment of DPN (14, 15) and analyzed its methodological, reporting, and evidence quality. However, the treatment interventions involve simple acupuncture, acupuncture + moxibustion, Chinese herbal medicine, and other traditional Chinese medicine (TCM) interventions. Moreover, with a separate analysis, the evidence provided may reflect the effect of acupuncture. Additionally, restricted by publication time, the reporting quality evaluation standard adopted is PRISMA2009, which has been updated to PRISMA2020. While literature retrieval ended in February 2020, multiple new MAs have been published. For the above reasons, AMSTAR-2 was adopted in this study to assess the methodological quality of MAs related to the acupuncture treatment of DPN. PRISMA2020 was employed to determine their reporting quality. This helped identify associated methodological problems and standardize the design, execution, and reporting of future relevant MAs. Based on a comprehensive summary of effectiveness and safety evidence, GRADE helped evaluate the evidence quality of outcome indicators, providing a basis for clinical decision-making and guideline development.

## 2 Data and methods

### 2.1 Retrieval strategies

Eight databases, including China National Knowledge Infrastructure (CNKI), Wanfang Data, CQVIP, CBM, PubMed, Cochrane Library, Embase, and Web of Science, were searched from database creation to December 22, 2023, to retrieve MAs associated with the acupuncture treatment of DPN. Retrieval strategies were developed with a combination of topic and free words. The main retrieval words included “DPN,” “painful diabetic neuropathy,” “acupuncture,” “electroacupuncture,” “blood-letting puncture,” “acupoint,” “auricular point,” “SR,” and “Meta-analysis.” The retrieval strategy for each database is represented in Supplementary material S1.

### 2.2 Literature inclusion and exclusion criteria

#### 2.2.1 Inclusion criteria

(1) Study type: MAs based on randomized controlled studies (RCTs). (2) Study subjects: patients diagnosed with DPN, with no limitations on age, gender, race, nationality, or disease course. (3) Interventions: The treatment group used acupuncture therapies or acupuncture therapies + conventional Western medicine. Acupuncture therapies involve filiform needle acupuncture, electroacupuncture, acupoint injection, acupoint patching, acupoint massage, and blood-letting puncture. The control group used conventional Western medicine alone. (4) Outcome indicators: total effective rate (TER), nerve conduction velocity (NCV), Toronto clinical scoring system (TCSS), visual analog scale (VAS), etc.

#### 2.2.2 Exclusion criteria

(1) Study protocols; (2) Network MAs, overviews of SRs, and qualitative SRs; (3) Animal experiments; (4) Literature related to conferences, newspapers, achievements, and change descriptions; (5) Duplicated publications, and literature with incomplete data or with no full text available.

### 2.3 Literature screening and data extraction

Two researchers independently conducted literature screening and compared the results. A third researcher would resolve any inconsistency or disagreement between their results. The literature screening process included three steps: Firstly, duplicate checking was performed using Notexpress Version 3.9.0.9640 to remove duplicated references; then, titles and abstracts were read for preliminary screening; finally, full texts were evaluated to determine the studies to be included.

A unified data extraction form was prepared using Excel and used by two researchers to extract data independently, followed by a result comparison. Any inconsistency or disagreement between their results would be resolved via discussion. The extracted data included the first author, publication year, searched databases, number of RCTs, total sample size, RCT quality evaluation tools, disease under investigation, treatment interventions, control interventions, outcome indicators, adverse events, and conclusions.

### 2.4 Methodological and reporting quality evaluation

Two researchers independently evaluated the methodological and reporting quality of the included studies. Any inconsistency or disagreement would be resolved through discussion. Methodological quality was assessed using the AMSTAR-2 scale (16). The AMSTAR-2 scale consisted of 16 items, including seven critical items (2, 4, 7, 9, 11, 13, 15) and nine non-critical items (1, 3, 5, 6, 8, 10, 12, 14, 16). Depending on the compliance of the included studies with relevant item standards, the evaluation results were judged as “Yes,” “Partial Yes,” or “No.” The overall quality of each SR was rated as “high,” “moderate,” “low,” or “critically low” by summing up these 16 items. The criteria are as follows: “high”: non-compliance of ≤1 non-critical item; “moderate”: non-compliance of >1 non-critical item; “low”: non-compliance of 1 critical item, with or without non-compliant non-critical items; “critically low”: non-compliance of >1 critical item, with or without non-compliant non-critical items.

The reporting quality of the included studies was evaluated using the PRISMA2020 (17) checklist, which has seven parts (i.e., title, abstract, introduction, methods, results, discussion, and other information) and 27 items (42 sub-items). Depending on the compliance of the included studies with relevant item standards, the evaluation results were judged as “Yes,” “Partial Yes,” or “No.”

### 2.5 Evidence quality evaluation

Two researchers independently evaluated the evidence quality of the outcome indicators of the included studies using GRADEpro GDT.^1^ Any inconsistency or disagreement would be resolved via discussion. The evidence quality of the MAs obtained from RCTs was initially rated as “high.” The primary concerns were the degradation factors, such as research limitations, heterogeneity, indirectness, imprecision, and publication bias. The final evidence quality of a single outcome indicator was “high,” “moderate,” “low,” or “critically low.” When there were overlapping outcome indicators between different MAs, their evidence quality was evaluated separately.

### 2.6 Statistical analysis

The basic characteristics of the included studies were described qualitatively. Descriptive statistical analysis was performed on the methodological and reporting quality of the included studies and the evidence quality of outcome indicators using Microsoft Excel 2021. The methodological quality evaluation results of the studies included were displayed using a bar chart, and a radar plot showed their reporting quality. The evidence quality and efficacy of outcome indicators were visualized from four dimensions using evidence mapping, *viz.*, (1) The abscissa represents evidence quality; (2) The ordinate represents outcome indicators; (3) The bubble area represents sample size; (4) The bubble color represents the *p*-value, with blue indicating *p* < 0.05 and orange *p* > 0.05.

## 3 Results

### 3.1 Results of literature screening

**Figure 1:**
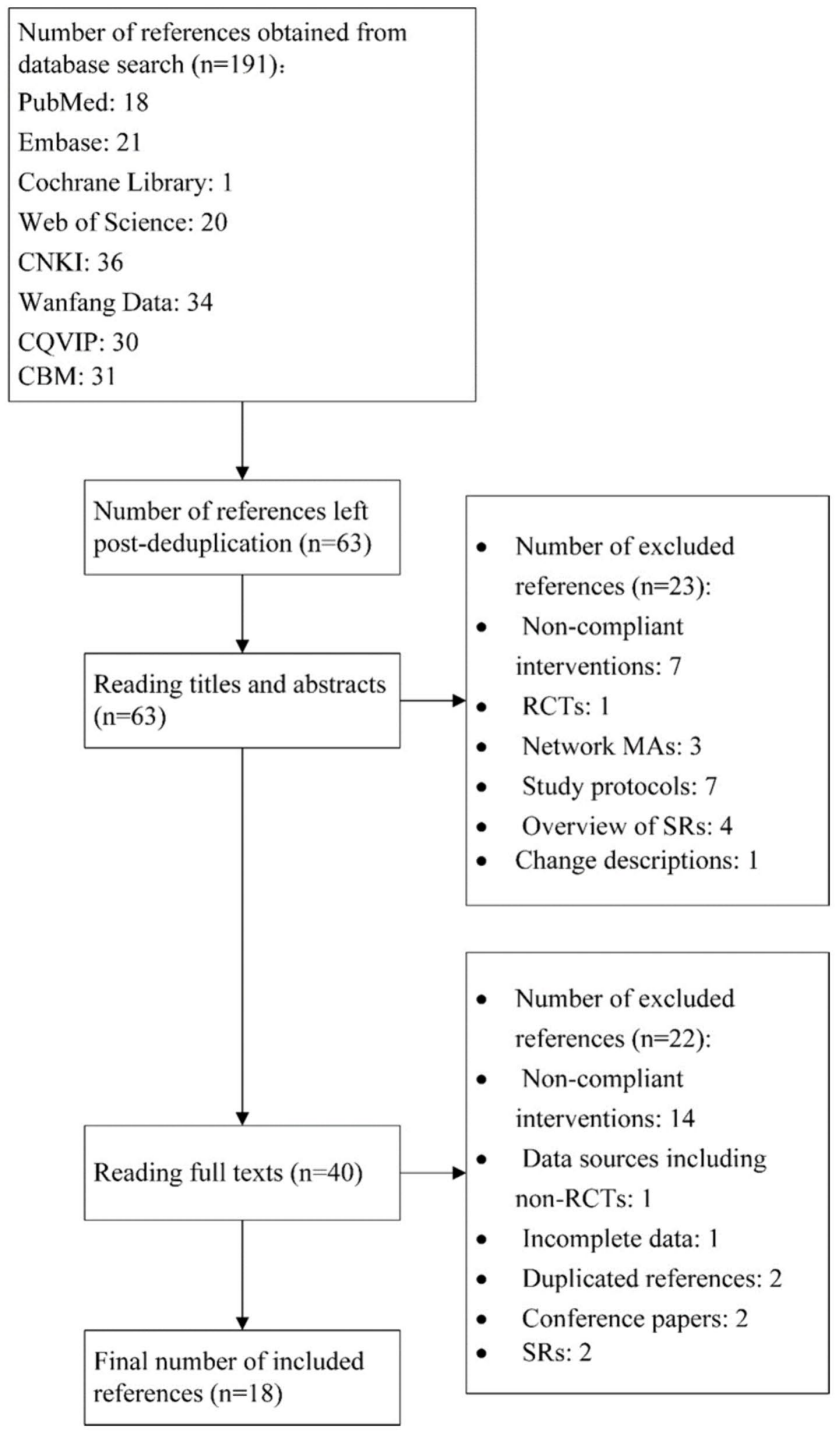
Literature screening process.

Initially, 191 references were obtained, and 63 were left after deduplication. Reading titles and abstracts helped exclude 23 references, and assessing full texts excluded another 22. Finally, 18 studies (18–35) were included. The literature screening process is represented in Figure 1.

### 3.2 Basic characteristics of the included studies

A total of 18 MAs (18–35) with 23,240 DPN patients were included. Of these, 17 (18, 20–35) involved DPN, and one (19) was linked with painful DPN. The most frequently searched English databases were PubMed, Cochrane Library, and Embase, and the most commonly searched Chinese databases included CNKI, Wanfang Data, CQVIP, and CBM. The minimum RCTS included was 2, and the maximum number was 69. The minimum and maximum sample sizes were 140 and 5,325, respectively. Thirteen studies (18–23, 25, 26, 28, 30–32, 34) used RoB as the RCT quality evaluation tool, four (24, 27, 29, 35) used Jadad, and one (33) did not mention it. Nine studies (18, 20–23, 25, 31, 35) used acupuncture therapies alone as treatment interventions, and 15 (19–21, 23–34) used acupuncture + conventional Western medicine. The outcome indicators primarily included TER, motor nerve conduction velocity (MNCV), or sensory nerve conduction velocity (SNCV) in the median nerve/common peroneal nerve/tibial nerve and TCSS. Four studies (19, 22, 25, 30) highlighted adverse events, primarily local pain. The basic characteristics of the included studies are represented in Supplementary material S2.

### 3.3 Methodological quality evaluation of the included studies

**Figure 2:**
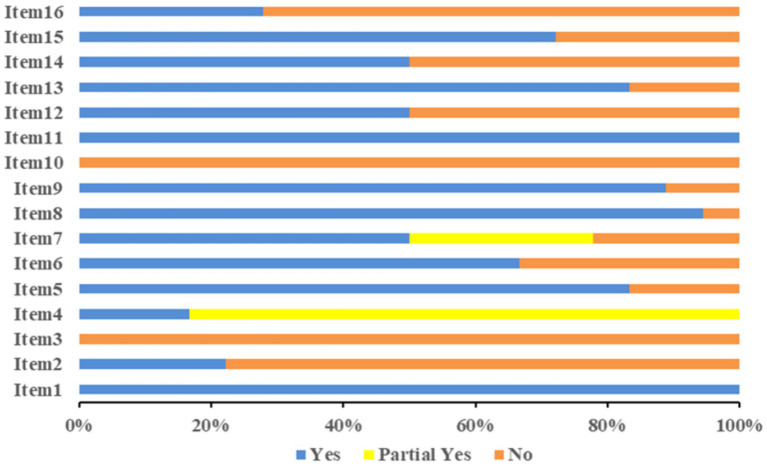
Methodological quality evaluation.

The methodological quality evaluation of the 18 studies obtained using AMSTAR-2 is shown in Figure 2. The compliance rates of items 2, 3, 10, 12, 14, and 16 were low (≤50%). The information on the protocol or registration (item 2) was not provided in 14 studies (21, 22, 24, 25). The study design inclusion (item 3) was not explained in any study (18–35). None of the studies reported funding (item 10) sources (18–35). The impact of the risk of bias of the studies in the results (item 12) was not considered in nine studies (24, 26, 27, 29, 30, 32–35). The heterogeneity between the studies (item 14) was not discussed in nine studies (20, 23, 24, 26, 27, 29, 32, 34, 35). No conflicts of interest (item 16) were reported in 13 studies (23–35). Among the 18 studies, four (18–20, 23) had a quality grade of “moderate,” seven (21, 22, 25, 26, 30–32) were “low,” and another seven (24, 27–29, 33–35) were “critically low.” The methodological quality evaluation of the studies is represented in Supplementary material S3.

### 3.4 Reporting quality evaluation of the included studies

**Figure 3:**
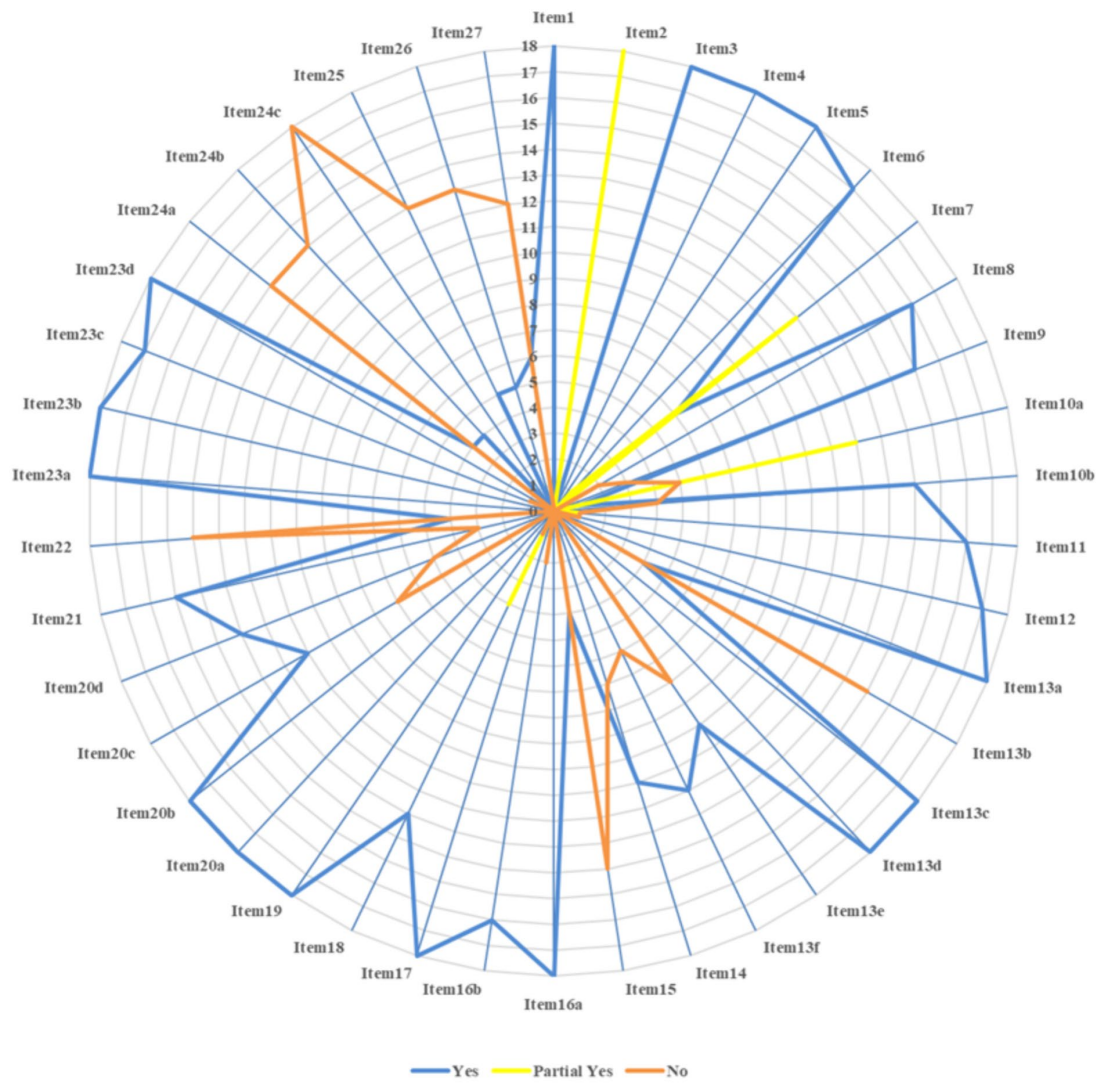
Reporting quality evaluation results.

The reporting quality evaluation of the 18 studies is represented in Figure 3. The compliance rates of items 2, 7, 10a, 13b, 15, 22, 24a, 24b, 24c, 25, 26, and 27 were low (≤50%). The abstract (item 2) was incomplete across 18 studies (18–35). Only retrieval words or the retrieval strategy for one database (item 7) was provided within 12 studies (21, 23–30, 33–35). Only the names of the outcome indicators extracted (item 10a) were mentioned within 12 studies (18–23, 25–27, 30, 31, 35) without elaborating on the measurement methods or time points. Five studies did not mention the extracted outcome indicators (24, 28, 29, 33, 34). Pre-processing before data consolidation (item 13b) was not mentioned in 14 studies (18, 20, 21, 23–30, 32, 33, 35). Neither evidence quality evaluation methods (item 15) nor results (item 22) were mentioned in 14 studies (20–30, 32, 33, 35). Registration information (item 24a) was not mentioned in 14 studies (21, 22, 24–35). The channel of access to the protocol (item 24b) was not mentioned in 14 studies (20–22, 24–30, 32–35). None of the studies described the modifications to the information on the protocol or registration (item 24c). Neither the funding sources for SRs (item 25) nor conflicts of interest (item 26) were mentioned in 13 studies (23–35). The channel of access to Supplementary material (item 27) was not mentioned in 12 studies (21, 24–30, 32–35). The reporting quality evaluation of the studies included is represented in Supplementary material S4.

### 3.5 Evidence quality evaluation of the included studies

The evidence quality of each outcome indicator of the studies was evaluated with GRADE. Among the outcome indicators of acupuncture vs. conventional Western medicine, four had a “moderate” evidence quality, 18 were “low,” and 17 were “critically low.” Among the outcome indicators of acupuncture + conventional Western medicine vs. conventional Western medicine, 12 had a “moderate” evidence quality, 29 were “low,” and 33 were “critically low.” The reasons for evidence quality degradation are as follows: (1) The RCTs included in the MAs had poor methodological quality, inducing a risk of bias. (2) The heterogeneity test obtained *I*^2^ > 50%, *p* < 0.01, causing inconsistency. (3) The sample size of binary variables was <300, and that of continuous variables was <400, giving rise to imprecision. (4) No English database was searched, and a small sample size with positive results led to a publication bias. The results and details of evidence quality evaluation by GRADE are provided in Supplementary material S5.

### 3.6 Efficacy evaluation

#### 3.6.1 Acupuncture vs. conventional Western medicine

**Figure 4:**
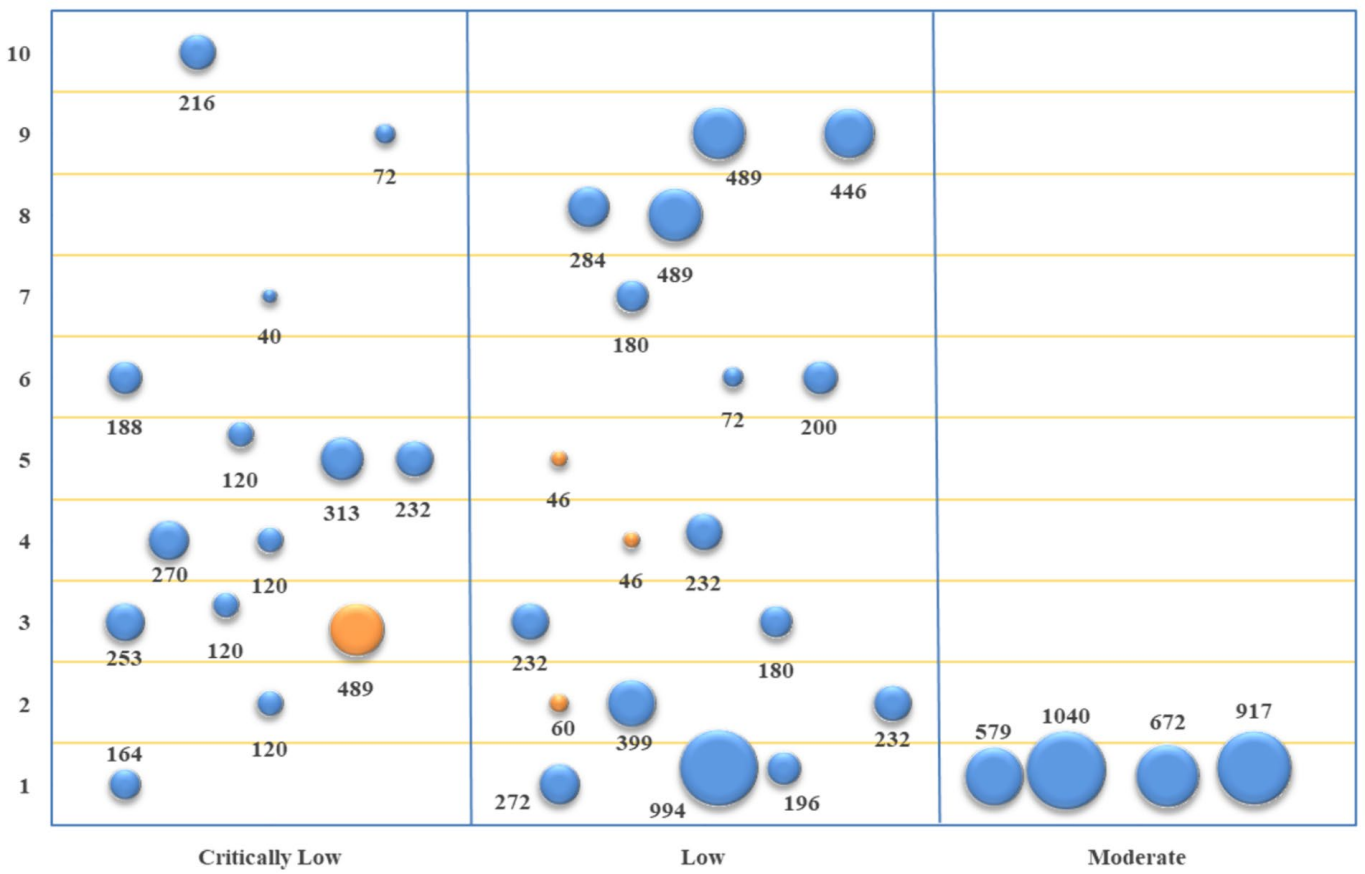
Evidence mapping of outcome indicators of acupuncture vs. conventional Western medicine. 1. TER; 2. SNCV in median nerve; 3. MNCV in median nerve; 4. SNCV in common peroneal nerve; 5. MNCV in common peroneal nerve; 6. SNCV in tibial nerve; 7. MNCV in tibial nerve; 8. SNCV in peroneal nerve; 9. MNCV in peroneal nerve; 10. TCSS.

The studies of acupuncture vs. conventional Western medicine involved 10 primary outcome indicators. The evidence quality, sample size, and *p*-value of each outcome indicator are depicted in Figure 4. According to the comparison between acupuncture and conventional Western medicine regarding improving TER, seven studies (18, 20, 21, 23, 25, 31, 35) and eight pieces of evidence indicated better performance of the acupuncture group (*p* < 0.05). In terms of improving SNCV in the median nerve, three studies (18, 25, 31) showed that the acupuncture group performed better (*p* < 0.05). In contrast, one study (20) revealed that acupuncture was equivalent to conventional Western medicine (*p* > 0.05). In terms of improving MNCV in the median nerve, four studies (18, 20, 31, 35) revealed that the acupuncture group performed better (*p* < 0.05). In contrast, one study (25) demonstrated that acupuncture was equivalent to conventional Western medicine (*p* > 0.05). In terms of improving SNCV in common peroneal nerve and MNCV in common peroneal nerve, three studies (18, 31, 35) showed that the acupuncture group performed better (*p* < 0.05). In contrast, one study (22) revealed that acupuncture was equivalent to conventional Western medicine (p > 0.05). In terms of improving SNCV in the tibial nerve, three studies (20, 22, 35) showed that the acupuncture group performed better (*p* < 0.05); in terms of improving MNCV in the tibial nerve, two studies (20, 22) showed that the acupuncture group performed better (*p* < 0.05). In terms of improving SNCV in the peroneal nerve, two studies (20, 25) showed that the acupuncture group performed better (*p* < 0.05); in terms of improving MNCV in the peroneal nerve, three studies (20, 22, 25) showed that the acupuncture group performed better (*p* < 0.05). In terms of improving TCSS, one study (25) showed that the acupuncture group performed better (*p* < 0.05).

#### 3.6.2 Acupuncture + conventional Western medicine vs. conventional Western medicine

**Figure 5:**
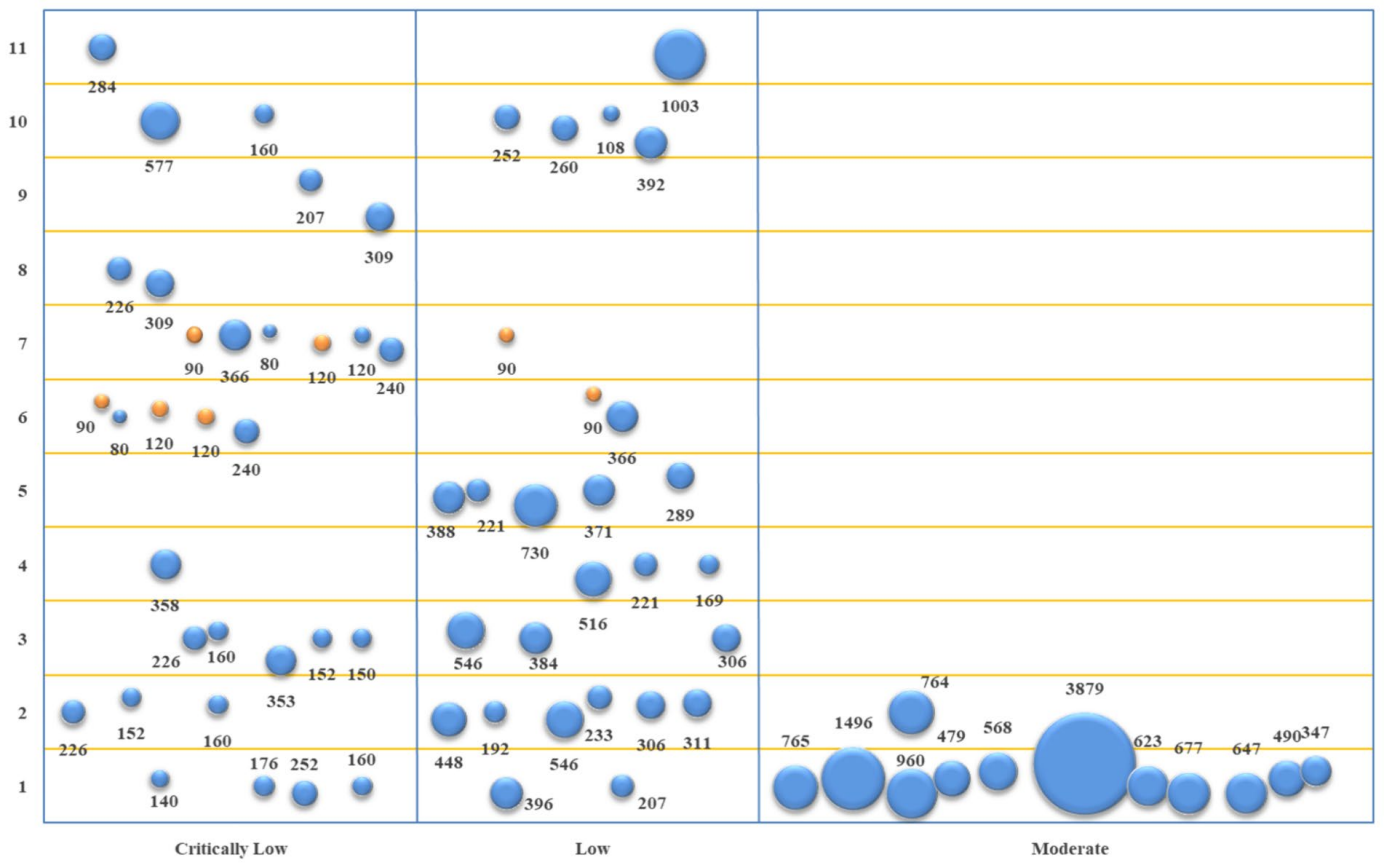
Evidence mapping of outcome indicators of acupuncture + conventional Western medicine vs. conventional Western medicine. 1. TER; 2. SNCV in median nerve; 3. MNCV in median nerve; 4. SNCV in common peroneal nerve; 5. MNCV in common peroneal nerve; 6. SNCV in tibial nerve; 7. MNCV in tibial nerve; 8. SNCV in peroneal nerve; 9. MNCV in peroneal nerve; 10. TCSS; 11. VAS.

The studies of acupuncture + conventional Western medicine vs. conventional Western medicine involved 11 primary outcome indicators. The evidence quality, sample size, and *p*-value of each outcome indicator are represented in Figure 5. Based on the comparison between acupuncture + conventional Western medicine and conventional Western medicine regarding improving TER, 14 studies (19–21, 23–32, 34) and 17 pieces of evidence revealed better performance of the combination group (*p* < 0.05). In terms of improving SNCV in the median nerve, eight studies (20, 25, 27, 28, 31–34) and 10 pieces of evidence indicated that the combination group performed better (*p* < 0.05). In terms of improving MNCV in the median nerve, seven studies (20, 25, 27, 28, 31, 33, 34) and eight pieces of evidence depicted that the combination group performed better (*p* < 0.05). In terms of improving SNCV in the common peroneal nerve, four studies (27, 31, 32, 34) showed that the combination group performed better (*p* < 0.05). In terms of improving MNCV in the common peroneal nerve, four studies (27, 31, 32, 34) and five pieces of evidence depicted that the combination group performed better (*p* < 0.05). In terms of improving SNCV in the tibial nerve, two studies (20, 33) and three pieces of evidence demonstrated that the combination group performed better (*p* < 0.05). In comparison, two studies (30, 32) and four pieces of evidence revealed equivalence between the combination group and the conventional Western medicine group (*p* > 0.05). In terms of improving MNCV in the tibial nerve, four studies (20, 30, 31, 33) showed that the combination group performed better (*p* < 0.05). In comparison, two studies (30, 32) and three pieces of evidence revealed that the combination group was equivalent to the conventional Western medicine group (*p* > 0.05). In terms of improving SNCV in the peroneal nerve and MNCV in the peroneal nerve, two studies (20, 25) showed that the combination group performed better (*p* < 0.05). In terms of improving TCSS, six studies (19, 20, 24, 26, 27, 31) showed that the combination group performed better (*p* < 0.05). In terms of improving VAS, one study (19) and two pieces of evidence depicted that the combination group performed better (*p* < 0.05).

## 4 Discussion

This study included 18 MAs of the acupuncture treatment of DPN and assessed their methodological quality with AMSTAR-2 and their reporting quality using PRISMA2020. The study also evaluated the evidence quality of outcome indicators using GRADE and summarized the efficacy of acupuncture treatment in DPN. Compared with conventional Western medicine, simple acupuncture, and acupuncture + conventional Western medicine had significant advantages in improving TER and NCV. The evidence quality of outcome indicators was low, and the methodological and reporting quality of relevant MAs requires further improvement.

### 4.1 Efficacy and safety of the acupuncture treatment of DPN

The common symptoms of DPN patients include numbness, pain, and paresthesia. Moreover, the common signs involve the weakening or disappearance of tendon reflex, vibration sensation, touch sensation, temperature sensation, acupuncture pain sensation, pressure sensation, etc. (36). TER usually reflects the improvement of symptoms and signs. NCV can reflect the severity of disease in DPN patients (36). This study showed that simple acupuncture and acupuncture + conventional Western medicine improved TER, along with NCV in the median nerve, common peroneal nerve, tibial nerve, etc. The mechanisms underlying the acupuncture treatment of DPN involve reducing the generation of spinal reactive oxygen species and alleviating oxidative phosphorylation (37), inhibiting glucose regulation protein 78 (GRP78, an endoplasmic reticulum chaperonin), relieving endoplasmic reticulum stress (38), and lowering P2X3, P2X4, and P2X7 receptor levels in spinal microglia (39–41). Acupuncture depends on these pathways to prevent nerve cell injury, improve NCV, and alleviate pain. Regarding safety, only four studies described that patients were intolerant of the discomfort induced by acupuncture or experienced local pain, with no other significant adverse reaction. Therefore, the acupuncture treatment of DPN is relatively safe and effective.

Existing evidence has illustrated the effectiveness of the acupuncture treatment of DPN. However, the GRADE evaluation showed low evidence quality in most cases, and most MAs indicated that multi-country, multi-center, large-sample, standardized RCTs and long-term follow-ups should further validate its effectiveness. The reasons are as follows: (1) The RCTs involved had low methodological quality, as embodied in defective randomization methods, allocation concealment, and the absence of a blinding method. (2) The lack of unified inclusion and exclusion criteria for study subjects induced baseline imbalance. (3) Quality of life, a key outcome indicator for DPN, did not receive adequate attention. (4) The definition of clinical efficacy was non-uniform and subjective to a certain degree. (5) The inconsistent methods for measuring and reporting outcome indicators limited evidence synthesis, necessitating the creation of a set of core outcome indicators for the RCTs of DPN. (6) Almost all RCTs were conducted in China. Hence, the DPN acupuncture treatment efficacy data does not apply to other countries.

### 4.2 Methodological quality of the included studies

Among the 18 included studies, only one had “moderate” methodological quality, while the majority were “low” or “critically low.” The compliance rates of items 2, 3, 10, 12, 14, and 16 were low. Among the 18 studies, only four completed protocol registration. Currently, the registration rates of SRs or MAs are low. A study surveyed 270 authors of SRs/MAs on their understanding of registration and observed that 44.2% of authors failed to complete protocol registration. This was due to their lack of awareness of protocol registration or their belief that the protocol might be leaked (42). In general, protocol registration can enhance the transparency of results, decrease duplication, and enhance the quality of SRs/MAs (43). Studies have shown a highly significant correlation between protocol registration and the impact factor of journals on which SRs/MAs are published (44). Therefore, future authors of SRs/MAs may pay close attention to protocol registration, and relevant registration agencies should strengthen protocol protection to safeguard the rights and interests of authors.

The study types included in the 18 MAs were all RCTs without reasonable explanation. Although RCTs are the gold standard for evaluating the clinical efficacy of interventions, there may be issues such as insufficient numbers, missing outcome indicators, unqualified study subjects, non-compliant interventions, and inadequate statistical efficacy. In such cases, non-RCTs can be included. This means that the study types included in SRs/MAs should not be arbitrary and should be selected based on real circumstances facilitated by a reasonable explanation (17).

None of the 18 studies reported the funding sources for primary studies. Information on the funding sources for primary studies is essential, as it helps to judge whether funding causes bias in SRs/MAs. Research has revealed that studies sponsored by pharmaceutical companies or medical device manufacturers are more likely to generate results favoring the sponsor (45). Therefore, authors of SRs/MAs should identify and report the funding sources for primary studies.

Nine of the 18 studies did not assess the impact of the risk of bias. If the RCTs included in SRs/MAs posed a low risk of bias, the results would be slightly affected. Since the quality of the RCTs included in 17 MAs varied, authors of MAs should investigate the potential impact of the risk of bias on the results (17).

Nine of the 18 studies failed to explain and discuss the impact of heterogeneity on the outcomes. The heterogeneity of SRs may originate from various sources, such as clinical, methodological, and statistical heterogeneity. The clinical heterogeneity of DPN-related MAs involved study populations, acupuncture points, acupuncture techniques, acupuncture treatment courses, control interventions, etc. The credibility of the synthesized results will be weakened without investigating and handling the sources of heterogeneity. For MAs with clinical and methodological heterogeneity, the sources of heterogeneity can be explored via subgroup analysis or Meta-regression. For those with statistical heterogeneity, a random effect model can be adapted to merge effect sizes, and sensitivity analysis can be used to evaluate the stability of the results. The impact of heterogeneity on the results has been elaborated in the discussion (46).

Only five of the 18 studies reported conflicts of interest. Without reporting conflicts of interest, it will be difficult for readers to judge whether there is a bias in the results of SRs/MAs. Projects funded by pharmaceutical companies are more likely to conclude effectiveness than other projects. Therefore, authors of SRs/MAs should describe the situation of funding, the role played by the sponsor in SRs (including whether they have participated in protocol development, execution, result analysis, writing, etc.), the way of handling conflicts of interest, etc. Without conflicts of interest, an explanation should also be provided (17).

### 4.3 Reporting quality of the included studies

According to the quality evaluation reports by PRISMA2020, the compliance rates of items 2, 7, 10a, 13b, 15, 22, 24a, 24b, 24c, 25, 26, and 27 were low. No comprehensive reporting of abstract information existed in any of the 18 studies. This is crucial since it increases information transparency and helps readers grasp the main contents of SRs and decide whether to download the full text (18). Only six of the 18 studies listed the retrieval strategy for each database. Reporting the retrieval strategy for each database enhances information transparency and helps evaluate the comprehensiveness and accuracy of retrieval strategies. Reporting SRs to validate the results also makes it convenient to update SRs. Only one of the 18 studies listed all outcome indicators that require data acquisition. The definition of an outcome indicator involves outcome-related events, measurement methods, time points, and analysis methods. Therefore, the primary studies included may report multiple results that meet the definition of SRs. Authors of SRs/MAs can extract all results that meet the definition of outcome indicators or select a specific subset of results (47). Authors should report relevant content so readers can judge whether there is a bias in the selection process. Only four of the 18 studies described the data preprocessing process. A detailed explanation of missing data processing and conversion helps readers comprehend the data preprocessing process and judge whether data synthesis is reasonable, thereby enhancing the transparency of SRs. Item 15 describes the method of evidence quality evaluation, and Item 22 characterizes the results of evidence quality evaluation. Four studies satisfied the relevant requirements in each case. Reporting the methods and results of evidence quality evaluation is a newly added content of PRISMA2020. This helps readers understand the standards of evidence evaluation and its credibility. Items 24a, 24b, and 24c report information on the protocol and registration and related changes. Only four studies reported information on the protocol and registration, and no study reported whether there were information changes. Reporting registration information helps to associate the publications of the same SR/MA in different journals. Reporting information on the protocol and related changes is helpful for readers to compare pre-designed contents using the final reported contents and assess whether any deviations pose a risk of bias (48). Without a protocol, an explanation should also be provided. Only five of the 18 studies reported funding sources and the role of the sponsor in SRs. This helps readers assess the impact of different funding types on SRs. Five studies reported conflicts of interest, which, as a newly added item of PRISMA2020, helps readers judge whether it impacts the results. Item 27 discloses data on SRs, with only six studies meeting relevant requirements. This allows readers to reuse the data, identify related issues, learn analytical methods, etc.

### 4.4 Advantages and limitations

The current study has the following advantages: Firstly, eight databases were searched to retrieve the MAs of DPN treatment by simple acupuncture and acupuncture + conventional Western medicine, summarizing the effectiveness and safety of treating DPN. Secondly, the methodological and reporting quality of the MAs included were evaluated using AMSTAR-2 and PRISMA2020. Thirdly, the quality of the evidence included was assessed using GRADE. Fourthly, the evidence quality and efficacy of each outcome indicator were represented by a bubble plot.

However, it is also important to point out some study limitations. Firstly, only Chinese and English literature was included, without including gray literature or studies in other languages. This could have affected the stability of the results, limiting generalization. Secondly, due to the heterogeneity of the MAs included in multiple aspects, including acupuncture techniques, needle retention time, treatment courses, and combined interventions, the primary studies of different outcome indicators were not merged, which could have also decreased the stability of the results.

## 5 Conclusion

Regarding DPN treatment, simple acupuncture and acupuncture + conventional Western medicine have provided significant advantages in improving TER and NCV with proven safety. However, the MAs of the acupuncture treatment of DPN should be improved in terms of methodological and reporting quality concerning AMSTAR-2 (16) and PRISMA2020 (17). The design, execution, and reporting of primary studies should follow CONSORT (49) to obtain higher-quality RCTs.

## Data Availability

The original contributions presented in the study are included in the article/Supplementary material, further inquiries can be directed to the corresponding authors.

## Footnotes

^1^https://gradepro.org/
