# Supplementary material for: Acupuncture treatment of diabetic peripheral neuropathy: an overview of systematic reviews based on evidence mapping
Source: Front Neurol. 2024 Oct 2;15:1420510. doi: 10.3389/fneur.2024.1420510 (PMC11483369; doi:10.3389/fneur.2024.1420510)
Supplement: Supplementary file 1 [file Table_1.DOCX]

Supplementary Material

# Retrieval strategy

## PubMed

("acupuncture"[Title/Abstract] OR "electroacupuncture"[Title/Abstract] OR "fire needle"[Title/Abstract] OR "scalp acupuncture"[Title/Abstract] OR "bloodletting therapy"[Title/Abstract] OR "acupoint"[Title/Abstract] OR "ear acupoint"[Title/Abstract]) AND ("meta-analysis"[Title] OR "systematic review"[Title]) AND ("diabetic peripheral neuropathy"[Title] OR "painful diabetic neuropathy"[Title])

## Embase

#1 'diabetic peripheral neuropathy':ti OR 'painful diabetic neuropathy':ti

#2 'meta analysis':ti OR 'systematic review':ti

#3 acupuncture:ab,ti OR electroacupuncture:ab,ti OR 'fire needle':ab,ti OR 'scalp acupuncture':ab,ti OR 'bloodletting therapy':ab,ti OR acupoint:ab,ti OR 'ear acupoint':ab,ti

#4 #1 AND #2 AND #3

## Cochrane Library

acupuncture OR electroacupuncture OR fire needle OR scalp acupuncture OR bloodletting therapy OR acupoint OR ear acupoint in Title Abstract Keyword AND diabetic peripheral neuropathy OR painful diabetic neuropathy in Title Abstract Keyword AND meta-analysis OR systematic review in Title Abstract Keyword

## Web of Science

((TI=(diabetic peripheral neuropathy)) AND TI=(meta-analysis OR systematic review)) AND AB=(acupuncture OR electroacupuncture OR fire needle OR scalp acupuncture OR bloodletting therapy OR acupoint OR ear acupoint)

## CNKI

TI='糖尿病周围神经病变' AND TKA=(‘针刺’+’针灸’+’电针’+’火针’+’头针’+’刺络放血’+’穴位’+’耳穴’) AND TI=('系统评价' + 'meta分析')

## Wanfang Data

TI=“糖尿病周围神经病变” AND AB=(“针刺”OR”针灸”OR”电针”OR”火针”OR”头针”OR”刺络放血”OR”穴位”OR”耳穴”) AND TI=(“系统评价” OR “meta分析”)

## CQVIP

T=糖尿病周围神经病变 AND R=(针刺OR针灸OR电针OR火针OR头针OR刺络放血OR穴位OR耳穴) AND T=(系统评价 OR meta分析)

## CBM

#1 “糖尿病周围神经病变”[标题]

#2 “系统评价”[标题] OR “meta分析”[标题]

#3 “针刺”[摘要] OR “针灸”[摘要] OR “电针”[摘要] OR “火针”[摘要] OR “头针”[摘要] OR “刺络放血”[摘要] OR “穴位”[摘要] OR “耳穴”[摘要]

#4 #1 AND #2 AND #3

# Basic characteristics of the included studies

| **Study** | **Retrieved databases** | **Number of RCTs** | **Total sample size** | **RCT quality evaluation tools** | **Disease under investigation** | **Treatment interventions** | **Control interventions** | **Outcome indicators** | **Adverse events** | **Conclusions** |
| --- | --- | --- | --- | --- | --- | --- | --- | --- | --- | --- |
| Zhang F2023 (18) | CNKI, Wanfang Data, CQVIP, CBM, PubMed, Cochrane Library, Embase, and Web of Science | 10 | 927 | RoB | DPN | Acupoint injection | Conventional Western medicine | A1, A4, A5, A7, A8 | Equivalent to the treatment group, not mentioned (NM) | Acupoint injection proven more effective |
| Zhou L2023 (19) | CNKI, Wanfang Data, CQVIP, CBM, PubMed, Cochrane Library, Embase, and Web of Science | 25 | 1,561 | RoB | Painful DPN | Acupuncture + conventional Western medicine | Conventional Western medicine | A16, A20 | Mild pain, swelling | Acupuncture or acupuncture + conventional Western medicine proven more effective |
| Jiang HL2020 (20) | CNKI, Wanfang Data, CQVIP, Medline, Web of Science, PubMed, Cochrane Library, Embase | 18 | 1,200 | RoB | DPN | Acupuncture/Acupuncture + conventional Western medicine | Conventional Western medicine | A1, A4, A5, A6, A9, A10, A11, A12, A16 | NM | Acupuncture or acupuncture + conventional Western medicine proven more effective |
| Xiong WJ2016 (21) | CNKI, Wanfang Data, CQVIP, CBM, China’s Important Conference Papers Database, China Dissertation Database (CDDB), Medline, CENTRAL | 12 | 837 | RoB | DPN | Acupuncture/Acupuncture + conventional Western medicine | Conventional Western medicine | A1 | NM | Acupuncture or acupuncture + conventional Western medicine proven more effective |
| Wang LQ 2018 (22) | CNKI, Wanfang Data, CQVIP, CBM, PubMed, Cochrane Library | 14 | 1,071 | RoB | DPN | Acupoint injection | Conventional Western medicine | A7, A8, A9, A10, A12, A13, A18, A19 | Local pain (the treatment group) | Acupuncture or acupuncture + conventional Western medicine proven more effective |
| Chen W2013 (23) | CNKI, CQVIP, CBM, China’s Important Conference Papers Database, CDDB, Cochrane Library, Medline, Embase | 25 | 1,649 | RoB | DPN | Acupuncture/Acupuncture + conventional Western medicine | Conventional Western medicine | A1 | NM | Acupoint injection + conventional Western medicine proven more effective |
| Gao J2021 (24) | CNKI, Wanfang Data, CQVIP, PubMed | 10 | 857 | Jadad | DPN | Acupoint patching + conventional Western medicine | Conventional Western medicine | A1, A16 | NM | Blood-letting puncture + conventional Western medicine proven more effective |
| Huang L2020 (25) | CNKI, Wanfang Data, CQVIP, CBM, PubMed, Embase, Cochrane Library | 15 | 1,153 | RoB | DPN | Acupuncture/Acupuncture + conventional Western medicine | Conventional Western medicine | A1, A4, A5, A11 A12, A16 | Dropout due to intolerance of pain from acupuncture (the treatment group) | Acupoint injection + conventional Western medicine proven more effective |
| Yang C2020 (26) | Wanfang Data, CQVIP, CBM, PubMed, manual retrieval of academic works and journal paper resources collected in the library of Changchun University of Chinese Medicine | 17 | 1,496 | RoB | DPN | Acupoint massage + conventional Western medicine | Conventional Western medicine | A1, A16 | NM | Acupuncture + conventional Western medicine proven more effective |
| He JB2019 (27) | CNKI, Wanfang Data, CQVIP, CBM, PubMed, Embase, Cochrane Library | 16 | 1,208 | Jadad | DPN | Acupuncture + conventional Western medicine/Blood-letting puncture + conventional Western medicine | Conventional Western medicine | A1, A2, A3, A4, A5, A7, A8, A16 | NM | Acupuncture + conventional Western medicine proven more effective |
| Zhu X2019 (28) | CNKI, Wanfang Data, CQVIP, Guizhou Digital Library, PubMed | 7 | 580 | RoB | DPN | Acupuncture + conventional Western medicine | Conventional Western medicine | A1, A4, A5 | NM | Acupoint patching + conventional Western medicine proven more effective |
| Sun HY2019 (29) | CNKI, CQVIP, PubMed, Cochrane Library | 2 | 140 | Jadad | DPN | Blood-letting puncture + conventional Western medicine | Conventional Western medicine | A1 | NM | Acupoint injection proven more effective |
| Yang XY2018 (30) | CNKI, Wanfang Data, CQVIP, CBM, Cochrane Library, Medline, AMED, CINAHL | 8 | 491 | RoB | DPN | Acupoint injection + conventional Western medicine | Conventional Western medicine | A1, A9, A10 | Dropout due to intolerance of Zusanli acupoint injection (the treatment group) | Acupuncture proven more effective |
| Chen Y2017 (31) | CNKI, Wanfang Data, CQVIP, CBM, PubMed, Cochrane Library, Embase, Duxiu (Slideum) | 25 | 1,900 | RoB | DPN | Acupuncture/Acupuncture + conventional Western medicine | Conventional Western medicine | A1, A4, A5, 7, A8, A9, A10, A16 | NM | Acupuncture or acupuncture + conventional Western medicine proven more effective |
| Zhu R2017 (32) | CNKI, Wanfang Data, CQVIP, CBM, Cochrane Library, Medline, Embase, AMED, CINAHL | 69 | 5,325 | RoB | DPN | Acupoint injection + conventional Western medicine/Acupoint massage + conventional Western medicine/Acupoint patching + conventional Western medicine | Conventional Western medicine | A1, A4, 7, A8, A9, A10, A14, A15 | NM | Acupuncture + conventional Western medicine proven more effective |
| Xu C2016 (33) | CNKI, Wanfang Data, CQVIP, CMB, PubMed | 7 | 546 | NM | DPN | Acupuncture + conventional Western medicine | Conventional Western medicine | A4, A5, A9, A10 | NM | Acupuncture or acupuncture + conventional Western medicine proven more effective |
| Li J2015 (34) | CNKI, Wanfang Data, Cochrane Library, Medline, Embase | 18 | 1,158 | RoB | DPN | Acupuncture + conventional Western medicine | Conventional Western medicine | A1, A4, A5, A7, A8 | NM | Acupuncture + conventional Western medicine proven more effective |
| Yang QW2014 (35) | CNKI, Wanfang Data, CBM | 16 | 1,141 | Jadad | DPN | Acupuncture | Conventional Western medicine | A1, A5, A7, A8, A9, A17 | NM | Acupoint massage + conventional Western medicine proven more effective |

A1= Total effective rate; A2= Symptom score; A3= Sign score; A4= Sensory nerve conduction velocity in median nerve; A5= Motor nerve conduction velocity in median nerve; A6= Motor nerve conduction velocity in ulnar nerve; A7= Sensory nerve conduction velocity in common peroneal nerve; A8= Motor nerve conduction velocity in common peroneal nerve; A9= Sensory nerve conduction velocity in tibial nerve; A10= Motor nerve conduction velocity in tibial nerve; A11= Sensory nerve conduction velocity in peroneal nerve; A12= Motor nerve conduction velocity in peroneal nerve; A13= Sensory nerve conduction velocity in superficial peroneal nerve; A14= Motor nerve conduction velocity in superficial peroneal nerve; A15= Sensory nerve conduction velocity in sural nerve; A16= Toronto clinical scoring system ; A17= NO; A18= Nerve conduction velocity in peroneal nerve; A19= Nerve conduction velocity in tibial nerve; A20= Visual analog scale

# The results of methodological quality evaluation

| **Study** | **1** | **2※** | **3** | **4※** | **5** | **6** | **7※** | **8** | **9※** | **10** | **11※** | **12** | **13※** | **14** | **15※** | **16** | **Ranking of quality** |
| --- | --- | --- | --- | --- | --- | --- | --- | --- | --- | --- | --- | --- | --- | --- | --- | --- | --- |
| Zhang F2023(18) | Y | Y | N | PY | Y | Y | Y | Y | Y | N | Y | Y | Y | Y | Y | Y | Moderate |
| Zhou L2023(19) | Y | Y | N | PY | Y | Y | Y | Y | Y | N | Y | Y | Y | Y | Y | Y | Moderate |
| Jiang HL2020(20) | Y | Y | N | PY | Y | Y | Y | Y | Y | N | Y | Y | Y | N | Y | Y | Moderate |
| Xiong WJ2016(21) | Y | N | N | Y | Y | Y | Y | Y | Y | N | Y | Y | Y | Y | Y | Y | Low |
| Wang LQ 2018(22) | Y | N | N | PY | Y | Y | Y | Y | Y | N | Y | Y | Y | Y | Y | Y | Low |
| Chen W 2013(23) | Y | Y | N | PY | Y | Y | Y | Y | Y | N | Y | Y | Y | N | Y | N | Moderate |
| Gao J2021(24) | Y | N | N | PY | N | N | PY | Y | Y | N | Y | N | N | N | N | N | Very low |
| Huang L2020(25) | Y | N | N | PY | Y | Y | Y | Y | Y | N | Y | Y | Y | Y | Y | N | Low |
| Yang C2020(26) | Y | N | N | PY | Y | N | PY | Y | Y | N | Y | N | Y | N | Y | N | Low |
| He JB2019(27) | Y | N | N | PY | Y | N | N | Y | N | N | Y | N | Y | N | Y | N | Very low |
| Zhu X2019(28) | Y | N | N | PY | Y | Y | PY | Y | Y | N | Y | Y | Y | Y | N | N | Very low |
| Sun HY2019(29) | Y | N | N | PY | N | Y | N | Y | Y | N | Y | N | N | N | N | N | Very low |
| Yang XY2018(30) | Y | N | N | PY | Y | Y | PY | Y | Y | N | Y | N | Y | Y | Y | N | Low |
| Chen Y2017(31) | Y | N | N | PY | Y | Y | Y | Y | Y | N | Y | Y | Y | Y | Y | N | Low |
| Zhu R2017(32) | Y | N | N | Y | Y | Y | Y | Y | Y | N | Y | N | Y | N | Y | N | Low |
| Xu C2016(33) | Y | N | N | PY | N | N | PY | Y | N | N | Y | N | N | Y | N | N | Very low |
| Li J2015(34) | Y | N | N | PY | Y | N | N | Y | Y | N | Y | N | Y | N | N | N | Very low |
| Yang QW2014(35) | Y | N | N | Y | Y | N | N | N | Y | N | Y | N | Y | N | Y | N | Very low |

※critical items; PY, Partial Yes; Y, Yes; N, No.

1. Did the research questions and inclusion criteria for the review include the components of PICO?

2. Did the report of the review contain an explicit statement that the review methods were established prior to the conduct of the review and did the report justify any significant deviations from the protocol?

3. Did the review authors explain their selection of the study designs for inclusion in the review?

4. Did the review authors use a comprehensive literature search strategy?

5. Did the review authors perform study selection in duplicate?

6. Did the review authors perform data extraction in duplicate?

7. Did the review authors provide a list of excluded studies and justify the exclusions?

8. Did the review authors describe the included studies in adequate detail?

9. Did the review authors use a satisfactory technique for assessing the risk of bias in individual studies that were included in the review?

10. Did the review authors report on the sources of funding for the studies included in the review?

11. If meta-analysis was performed did the review authors use appropriate methods for statistical combination of results?

12. If meta-analysis was performed, did the review authors assess the potential impact of risk of bias in individual studies on the results of the meta-analysis or other evidence synthesis?

13. Did the review authors account for risk of bias in individual studies when interpreting/ discussing the results of the review?

14. Did the review authors provide a satisfactory explanation for, and discussion of, any heterogeneity observed in the results of the review?

15. If they performed quantitative synthesis did the review authors carry out an adequate investigation of publication bias (small study bias) and discuss its likely impact on the results of the review?

16. Did the review authors report any potential sources of conflict of interest, including any funding they received for conducting the review?

# The results of reporting quality evaluation

| Part | Item | Zhang F2023(18) | Zhou L2023(19) | Jiang HL2020(20) | Xiong WJ2016(21) | Wang LQ 2018(22) | Chen W 2013(23) | Gao J2021(24) | Huang L2020(25) | Yang C2020(26) | He JB2019(27) | Zhu X2019(28) | Sun HY2019(29) | Yang XY2018(30) | Chen Y2017(31) | Zhu R2017(32) | Xu C2016(33) | Li J2015(34) | Yang QW2014(35) |
| --- | --- | --- | --- | --- | --- | --- | --- | --- | --- | --- | --- | --- | --- | --- | --- | --- | --- | --- | --- |
| Title | 1 | Y | Y | Y | Y | Y | Y | Y | Y | Y | Y | Y | Y | Y | Y | Y | Y | Y | Y |
| Abstract | 2 | PY | PY | PY | PY | PY | PY | PY | PY | PY | PY | PY | PY | PY | PY | PY | PY | PY | PY |
| Introduction | 3 | Y | Y | Y | Y | Y | Y | Y | Y | Y | Y | Y | Y | Y | Y | Y | Y | Y | Y |
|  | 4 | Y | Y | Y | Y | Y | Y | Y | Y | Y | Y | Y | Y | Y | Y | Y | Y | Y | Y |
| Methods | 5 | Y | Y | Y | Y | Y | Y | Y | Y | Y | Y | Y | Y | Y | Y | Y | Y | Y | Y |
|  | 6 | Y | Y | Y | Y | Y | Y | Y | Y | Y | Y | Y | PY | Y | Y | Y | Y | Y | Y |
|  | 7 | Y | Y | Y | PY | Y | PY | PY | PY | PY | PY | PY | PY | PY | Y | Y | PY | PY | PY |
|  | 8 | Y | Y | Y | Y | Y | Y | N | Y | Y | Y | Y | Y | Y | Y | Y | N | Y | Y |
|  | 9 | Y | Y | Y | Y | Y | Y | N | Y | Y | Y | Y | Y | Y | Y | Y | N | N | Y |
|  | 10a | PY | PY | PY | PY | PY | PY | N | PY | PY | PY | N | N | PY | PY | Y | N | N | PY |
|  | 10b | Y | Y | Y | Y | Y | Y | N | Y | Y | Y | N | Y | Y | Y | Y | N | N | Y |
|  | 11 | Y | Y | Y | Y | Y | Y | Y | Y | PY | Y | Y | Y | Y | Y | Y | N | Y | Y |
|  | 12 | Y | Y | Y | Y | Y | Y | Y | Y | Y | Y | Y | Y | Y | Y | Y | N | Y | Y |
|  | 13a | Y | Y | Y | Y | Y | Y | Y | Y | Y | Y | Y | Y | Y | Y | Y | Y | Y | Y |
|  | 13b | N | Y | N | N | Y | N | N | N | N | N | N | N | N | Y | N | N | Y | N |
|  | 13c | Y | Y | Y | Y | Y | Y | Y | Y | Y | Y | Y | Y | Y | Y | Y | Y | Y | Y |
|  | 13d | Y | Y | Y | Y | Y | Y | Y | Y | Y | Y | Y | Y | Y | Y | Y | Y | Y | Y |
|  | 13e | Y | Y | Y | Y | N | N | N | Y | N | N | Y | N | Y | Y | Y | N | Y | N |
|  | 13f | Y | Y | Y | Y | N | Y | N | Y | N | Y | Y | Y | Y | Y | Y | N | N | N |
|  | 14 | Y | Y | Y | Y | N | Y | N | Y | N | Y | N | Y | N | Y | Y | N | N | Y |
|  | 15 | Y | Y | N | N | N | N | N | N | N | N | N | N | N | Y | N | N | Y | N |
| Results | 16a | Y | Y | Y | Y | Y | Y | Y | Y | Y | Y | Y | Y | Y | Y | Y | Y | Y | Y |
|  | 16b | Y | Y | Y | Y | Y | Y | Y | Y | Y | Y | Y | N | Y | Y | Y | Y | Y | N |
|  | 17 | Y | Y | Y | Y | Y | Y | Y | Y | Y | Y | Y | Y | Y | Y | Y | Y | Y | Y |
|  | 18 | Y | Y | Y | Y | Y | Y | PY | Y | Y | PY | Y | PY | Y | Y | Y | N | Y | PY |
|  | 19 | Y | Y | Y | Y | Y | Y | Y | Y | Y | Y | Y | Y | Y | Y | Y | Y | Y | Y |
|  | 20a | Y | Y | Y | Y | Y | Y | Y | Y | Y | Y | Y | Y | Y | Y | Y | Y | Y | Y |
|  | 20b | Y | Y | Y | Y | Y | Y | Y | Y | Y | Y | Y | Y | Y | Y | Y | Y | Y | Y |
|  | 20c | Y | Y | Y | Y | Y | N | N | Y | N | N | Y | Y | N | Y | Y | N | Y | N |
|  | 20d | Y | Y | Y | Y | Y | Y | N | Y | N | Y | Y | Y | N | Y | Y | N | Y | N |
|  | 21 | Y | Y | Y | Y | Y | Y | Y | Y | Y | Y | N | Y | Y | Y | Y | N | N | Y |
|  | 22 | Y | Y | N | N | N | N | N | N | N | N | N | N | N | Y | N | N | Y | N |
| Discussion | 23a | Y | Y | Y | Y | Y | Y | Y | Y | Y | Y | Y | Y | Y | Y | Y | Y | Y | Y |
|  | 23b | Y | Y | Y | Y | Y | Y | Y | Y | Y | Y | Y | Y | Y | Y | Y | Y | Y | Y |
|  | 23c | Y | Y | Y | Y | Y | Y | Y | Y | Y | Y | Y | Y | Y | Y | Y | N | Y | Y |
|  | 23d | Y | Y | Y | Y | Y | Y | Y | Y | Y | Y | Y | Y | Y | Y | Y | Y | Y | Y |
| Other Information | 24a | Y | Y | Y | N | N | Y | N | N | N | N | N | N | N | N | N | N | N | N |
|  | 24b | Y | Y | N | N | N | Y | N | N | N | N | N | N | N | Y | N | N | N | N |
|  | 24c | N | N | N | N | N | N | N | N | N | N | N | N | N | N | N | N | N | N |
|  | 25 | Y | Y | Y | Y | Y | N | N | N | N | N | N | N | N | N | N | N | N | N |
|  | 26 | Y | Y | Y | Y | Y | N | N | N | N | N | N | N | N | N | N | N | N | N |
|  | 27 | Y | Y | Y | N | Y | Y | N | N | N | N | N | N | N | Y | N | N | N | N |

Item 1: Title; Item 2: Abstract; Item 3: Rationale; Item 4: Objectives; Item 5: Eligibility criteria; Item 6: Information sources; Item 7: Search strategy; Item 8: Selection process; Item 9: Data collection process; Item 10a: Data items; Item 11: Study risk of bias assessment; Item 12: Effect measures; Item 13: Synthesis methods; Item 14: Reporting bias assessment; Item 15: Certainty assessment; Item 16: Study selection; Item 17: Study characteristics; Item 18: Risk of bias in studies; Item 19: Results of individual studies; Item 20: Results of syntheses; Item 21: Reporting biases; Item 22: Certainty of evidence; Item 23: Discussion; Item 24: Registration and protocol; Item 25: Support; Item 26: Competing interests; Item 27: Availability of data, code and other materials.

# The results of evidence quality evaluation by GRADE

## The results of evidence quality evaluation by GRADE（Acupuncture vs. conventional Western medicine）

| Outcomes | Study | Efect Size (95% CI) | *P* | I^2^(%) | Risk of bias | Inconsistency | Indirectness | Imprecision | Publication bias | GRADE quality |
| --- | --- | --- | --- | --- | --- | --- | --- | --- | --- | --- |
| Total effective rate | Huang L2020(25) | RR:0.39(0.29, 0.54) | <0.00001 | 0 | serious^a^ | not serious | not serious | not serious | none | Moderate |
|  | Chen Y2017(31) | RR:1.36(1.18, 1.56) | <0.0001 | 0 | serious^a^ | not serious | not serious | serious^e^ | none | Low |
|  | Chen W2013(23) | RR:1.37(1.27, 1.46) | <0.00001 | 9 | serious^a^ | not serious | not serious | not serious | none | Moderate |
|  | Jiang HL2020(20) | RR:1.32(1.21, 1.43) | <0.00001 | 0 | serious^a^ | not serious | not serious | not serious | none | Moderate |
|  | Yang QW2014(35) | OR:5.51(3.87, 7.83) | <0.00001 | 3 | serious^a^ | not serious | not serious | not serious | publication bias strongly suspected^f^ | Low |
|  | Zhang F2023(18) | RR:1.27(1.19, 1.36) | <0.00001 | 0 | serious^a^ | not serious | not serious | not serious | none | Moderate |
|  | Xiong WJ2016(21) | RR:1.29(1.14, 1.46) | <0.0001 | 0 | serious^a^ | not serious | not serious | serious^e^ | none | Low |
|  | Xiong WJ2016(21) | RR:1.60(1.33, 1.94) | <0.00001 | 0 | serious^a^ | not serious | not serious | serious^e^ | publication bias strongly suspected^c^ | Very low |
| SNCV in median nerve | Huang L2020(25) | SMD:0.56(0.08, 1.03) | <0.0001 | 82 | serious^a^ | serious^d^ | not serious | not serious | none | Low |
|  | Chen Y2017(31) | SMD:0.73(0.36, 1.1) | <0.0001 | 22 | serious^a^ | not serious | not serious | serious^b^ | publication bias strongly suspected^c^ | Very low |
|  | Jiang HL2020(20) | MD:0.94(1.05, 2.93) | 0.36 | - | serious^a^ | not serious | not serious | serious^b^ | none | Low |
|  | Zhang F2023(18) | MD:4.83(3.75, 5.9) | <0.00001 | 40 | serious^a^ | not serious | not serious | serious^b^ | none | Low |
| MNCV in median nerve | Huang L2020(25) | SMD:0.5(0.31, 1.3) | 0.22 | 94 | serious^a^ | serious^d^ | not serious | not serious | none | Low |
|  | Chen Y2017(31) | SMD:1(0.61, 1.38) | <0.00001 | 0 | serious^a^ | not serious | not serious | serious^b^ | publication bias strongly suspected^c^ | Very low |
|  | Jiang HL2020(20) | MD:3.07(1.92, 4.21) | <0.00001 | 0 | serious^a^ | not serious | not serious | serious^b^ | none | Low |
|  | Yang QW2014(35) | MD:2.02(0.18, 3.36) | 0.03 | 57 | serious^a^ | not serious | not serious | serious^b^ | publication bias strongly suspected^f^ | Very low |
|  | Zhang F2023(18) | MD:5.93(4.79, 7.07) | 0.26 | 26 | serious^a^ | not serious | not serious | serious^b^ | none | Low |
| SNCV in common peroneal nerve | Chen Y2017(31) | SMD:1.35(0.95, 1.75) | <0.00001 | 0 | serious^a^ | not serious | not serious | serious^b^ | publication bias strongly suspected^c^ | Very low |
|  | Wang LQ 2018(22) | MD:1.43(1.41, 4.27) | 0.32 | - | serious^a^ | not serious | not serious | serious^b^ | none | Low |
|  | Yang QW2014(35) | MD:2.02(0.18, 3.36) | <0.00001 | 30 | serious^a^ | not serious | not serious | serious^b^ | publication bias strongly suspected^f^ | Very low |
|  | Zhang F2023(18) | MD:3.6(2.49, 4.71) | <0.00001 | 0 | serious^a^ | not serious | not serious | serious^b^ | none | Low |
| MNCV in common peroneal nerve | Chen Y2017(31) | SMD:1.05(0.57, 1.44) | <0.00001 | 50 | serious^a^ | serious^d^ | not serious | serious^b^ | publication bias strongly suspected^c^ | Very low |
|  | Wang LQ 2018(22) | MD:0.67(2.37, 3.71) | 0.67 | - | serious^a^ | not serious | not serious | serious^b^ | none | Low |
|  | Yang QW2014(35) | MD:4.15(0.53, 7.77) | <0.00001 | 95 | serious^a^ | serious^d^ | not serious | serious^b^ | publication bias strongly suspected^f^ | Very low |
|  | Zhang F2023(18) | MD:5.66(2.89, 8.43) | 0.001 | 85 | serious^a^ | serious^d^ | not serious | serious^b^ | none | Very low |
| SNCV in tibial nerve | Wang LQ 2018(22) | MD:2.84(0.46, 5.22) | 0.02 | - | serious^a^ | not serious | not serious | serious^b^ | none | Low |
|  | Jiang HL2020(20) | MD:1.73(0.98, 2.48) | <0.00001 | 0 | serious^a^ | not serious | not serious | serious^b^ | none | Low |
|  | Yang QW2014(35) | MD:1.68(1.05, 2.32) | <0.00001 | 0 | serious^a^ | not serious | not serious | serious^b^ | publication bias strongly suspected^f^ | Very low |
| MNCV in tibial nerve | Wang LQ 2018(22) | MD:9.4(6.19, 12.61) | <0.01 | - | serious^a^ | not serious | not serious | serious^b^ | publication bias strongly suspected^c^ | Very low |
|  | Jiang HL2020(20) | MD:2.58(1.71, 3.44) | <0.00001 | 2 | serious^a^ | not serious | not serious | serious^b^ | none | Low |
| SNCV in peroneal nerve | Huang L2020(25) | SMD:0.77(0.19, 1.34) | <0.00001 | 89 | serious^a^ | serious^d^ | not serious | not serious | none | Low |
|  | Jiang HL2020(20) | MD:3.97(2.85, 5.08) | <0.00001 | 0 | serious^a^ | not serious | not serious | serious^b^ | none | Low |
| MNCV in peroneal nerve | Huang L2020(25) | SMD:1(0.69, 1.31) | <0.00001 | 61 | serious^a^ | serious^d^ | not serious | not serious | none | Low |
|  | Wang LQ 2018(22) | MD:0.67(2.37, 3.71) | 0.67 | - | serious^a^ | not serious | not serious | serious^b^ | none | Low |
|  | Jiang HL2020(20) | MD:4.79(2.46, 7.13) | <0.00001 | 88 | serious^a^ | serious^d^ | not serious | not serious | none | Low |
| SNCV in superficial peroneal nerve | Wang LQ 2018(22) | MD:7.83(5.02, 10.64) | <0.01 | - | serious^a^ | not serious | not serious | serious^b^ | publication bias strongly suspected^c^ | Very low |
| MNCV in ulnar nerve | Jiang HL2020(20) | MD:11.32(9.44, 13.2) | <0.00001 | - | serious^a^ | not serious | not serious | serious^b^ | publication bias strongly suspected^c^ | Very low |
| TCSS | Huang L2020(25) | SMD:1.62(2.21, 1.03) | <0.00001 | 69 | serious^a^ | not serious | not serious | not serious | none | Moderate |
| NO | Yang QW2014(35) | MD:6.15(1.84, 10.46) | 0.005 | 74 | serious^a^ | serious^d^ | not serious | serious^b^ | publication bias strongly suspected^c,f^ | Very low |

Explanations:

a. The RCTs included in the MAs had poor methodological quality, inducing a risk of bias.

b. The sample size of continuous variables was <400.

c. A small sample size with positive results.

d. The heterogeneity test obtained I^2^＞50%, P<0.01.

e. The sample size of binary variables was <300.

f. No English database was searched.

## The results of evidence quality evaluation by GRADE（Acupuncture + Conventional Western medicine vs. Conventional Western medicine）

| Outcomes | Study | Efect Size (95% CI) | *P* | I^2^(%) | Risk of bias | Inconsistency | Indirectness | Imprecision | Publication bias | GRADE quality |
| --- | --- | --- | --- | --- | --- | --- | --- | --- | --- | --- |
| Total effective rate | Huang L2020(25) | RR:0.33(0.18, 0.61) | 0.0005 | 0 | serious^a^ | not serious | not serious | serious^b^ | none | Low |
|  | Gao J2021(24) | OR:4.45(2.95, 6.70) | <0.00001 | 0 | serious^a^ | not serious | not serious | not serious | none | Moderate |
|  | Yang C2020(26) | OR:5.84(4.32, 7.90) | <0.00001 | 30 | serious^a^ | not serious | not serious | not serious | none | Moderate |
|  | He JB2019(27) | RR:1.14(1.03, 1.27) | <0.0001 | 0 | serious^a^ | not serious | not serious | not serious | none | Moderate |
|  | Zhu X2019(28) | OR:3.85(2.34, 6.35) | <0.00001 | 7 | serious^a^ | not serious | not serious | not serious | none | Moderate |
|  | Sun HY2019(29) | OR: 3.82(1.51, 9.70) | 0.005 | 0 | serious^a^ | not serious | not serious | serious^e^ | publication bias strongly suspected^d^ | Very low |
|  | Chen Y2017(31) | RR:1.34(1.23, 1.46) | <0.00001 | 24 | serious^a^ | not serious | not serious | not serious | none | Moderate |
|  | Zhu R2017(32) | OR:4.51(3.80, 5.37) | <0.00001 | 19 | serious^a^ | not serious | not serious | not serious | none | Moderate |
|  | Zhu R2017(32) | OR:7.27(4.44, 11.90) | <0.00001 | 12 | serious^a^ | not serious | not serious | not serious | none | Moderate |
|  | Zhu R2017(32) | OR:4.03(2.64, 6.17) | <0.00001 | 0 | serious^a^ | not serious | not serious | not serious | none | Moderate |
|  | Li J2015(34) | RR:1.48(1.29, 1.70) | <0.00001 | 47 | serious^a^ | not serious | not serious | not serious | none | Moderate |
|  | Chen W 2013(23) | RR:1.56(1.28, 1.90) | <0.0001 | 75 | serious^a^ | serious^c^ | not serious | serious^e^ | none | Very low |
|  | Jiang HL2020(20) | RR:1.44(1.10, 1.89) | 0.009 | 71 | serious^a^ | serious^c^ | not serious | serious^b^ | none | Very low |
|  | Yang XY2018(30) | OR:3.46(2.07, 5.77) | <0.00001 | 23 | serious^a^ | not serious | not serious | not serious | none | Moderate |
|  | Chen Y2017(31) | RR:1.33(1.19, 1.48) | <0.00001 | 26 | serious^a^ | not serious | not serious | not serious | none | Moderate |
|  | Zhou L2023(19) | RR:1.39(1.21, 1.59) | <0.00001 | 0 | serious^a^ | not serious | not serious | serious^e^ | none | Low |
|  | Xiong WJ2016(21) | RR:1.20(1.03, 1.40) | <0.00001 | 33 | serious^a^ | not serious | not serious | serious^e^ | publication bias strongly suspected^d^ | Very low |
| Symptom score | He JB2019(27) | MD:3.39(3.46, 3.32) | <0.0001 | 0 | serious^a^ | not serious | not serious | serious^b^ | none | Low |
| Sign score | He JB2019(27) | MD:1.81(1.88, 1.75) | <0.0001 | 0 | serious^a^ | not serious | not serious | serious^b^ | none | Low |
| SNCV in median nerve | Huang L2020(25) | SMD:2.28(0.61, 3.96) | <0.00001 | 96 | serious^a^ | serious^c^ | not serious | serious^b^ | none | Very low |
|  | He JB2019(27) | MD:3.17(2.48, 3.86) | <0.0001 | 0 | serious^a^ | not serious | not serious | serious^b^ | none | Low |
|  | Zhu X2019(28) | MD:6.2(4.49, 7.91) | <0.00001 | 0 | serious^a^ | not serious | not serious | serious^b^ | publication bias strongly suspected^d^ | Very low |
|  | Chen Y2017(31) | SMD:0.48(0.17, 0.8) | 0.003 | 20 | serious^a^ | not serious | not serious | serious^b^ | publication bias strongly suspected^d^ | Very low |
|  | Zhu R2017(32) | MD:4.06(3.47, 4.64) | <0.00001 | 20 | serious^a^ | not serious | not serious | not serious | none | Moderate |
|  | Zhu R2017(32) | MD:1.17(0.46, 1.89) | 0.001 | 33 | serious^a^ | not serious | not serious | serious^b^ | none | Low |
|  | Xu C2016(33) | MD:4.27(1.94, 6.6) | 0.0003 | 90 | serious^a^ | serious^c^ | not serious | not serious | none | Low |
|  | Li J2015(34) | MD:2.11(1.01, 3.21) | 0.0002 | 34 | serious^a^ | not serious | not serious | serious^b^ | none | Low |
|  | Jiang HL2020(20) | MD:3.42(2.33, 4.51) | <0.00001 | 0 | serious^a^ | not serious | not serious | serious^b^ | none | Low |
|  | Chen Y2017(31) | SMD:0.78(0.55, 1.02) | <0.00001 | 27 | serious^a^ | not serious | not serious | serious^b^ | none | Low |
| MNCV in median nerve | Huang L2020(25) | SMD:2.33(1.19, 3.47) | <0.0001 | 91 | serious^a^ | serious^c^ | not serious | serious^b^ | none | Very low |
|  | He JB2019(27) | MD:4.56(3.97, 5.15) | <0.0001 | 21 | serious^a^ | not serious | not serious | serious^b^ | none | Low |
|  | Chen Y2017(31) | SMD:0.6(0.29, 0.92) | 0.0002 | 0 | serious^a^ | not serious | not serious | serious^b^ | publication bias strongly suspected^d^ | Very low |
|  | Xu C2016(33) | MD:6.66(3.66, 9.65 higher) | <0.00001 | 93 | serious^a^ | serious^c^ | not serious | not serious | none | Low |
|  | Li J2015(34) | MD:4.37(0.77, 7.98) | <0.00001 | 94 | serious^a^ | serious^c^ | not serious | serious^b^ | none | Very low |
|  | Jiang HL2020(20) | MD:5.43(3.27, 7.6) | <0.00001 | 69 | serious^a^ | not serious | not serious | serious^b^ | none | Low |
|  | Zhu X2019(28) | MD:6.18(4.64, 7.72) | <0.00001 | 0 | serious^a^ | not serious | not serious | serious^b^ | publication bias strongly suspected^d^ | Very low |
|  | Chen Y2017(31) | SMD:1.37(0.96, 1.78) | <0.00001 | 0 | serious^a^ | not serious | not serious | serious^b^ | publication bias strongly suspected^d^ | Very low |
| SNCV in common peroneal nerve | He JB2019(27) | MD:3.47(2.99, 3.95) | <0.0001 | 76.5 | serious^a^ | serious^c^ | not serious | not serious | none | Low |
|  | Chen Y2017(31) | SMD:0.72(0.45, 1) | <0.00001 | 14 | serious^a^ | not serious | not serious | serious^b^ | none | Low |
|  | Zhu R2017(32) | MD:4.5(3.13, 5.87) | <0.00001 | 76 | serious^a^ | serious^c^ | not serious | serious^b^ | none | Very low |
|  | Li J2015(34) | MD:3.29(2.3, 4.28) | <0.00001 | 0 | serious^a^ | not serious | not serious | serious^b^ | none | Low |
| MNCV in common peroneal nerve | He JB2019(27) | MD:4.58(3.86, 5.31) | <0.00001 | 48.2 | serious^a^ | not serious | not serious | serious^b^ | none | Low |
|  | Chen Y2017(31) | SMD:0.63(0.36, 0.9) | <0.00001 | 0 | serious^a^ | not serious | not serious | serious^b^ | none | Low |
|  | Zhu R2017(32) | MD:3.88(2.32, 5.44) | <0.00001 | 82 | serious^a^ | serious^c^ | not serious | not serious | none | Low |
|  | Zhu R2017(32) | MD:2.47(1.79, 3.16) | <0.00001 | 0 | serious^a^ | not serious | not serious | serious^b^ | none | Low |
|  | Li J2015(34) | MD:3.34(2.18, 4.51) | <0.00001 | 48 | serious^a^ | not serious | not serious | serious^b^ | none | Low |
| SNCV in tibial nerve | Zhu R2017(32) | MD:1.49(0.43, 3.4) | 0.13 | 0 | serious^a^ | not serious | not serious | serious^b^ | none | Low |
|  | Zhu R2017(32) | MD:1.72(5.42, 1.99) | 0.36 | 80 | serious^a^ | serious^c^ | not serious | serious^b^ | none | Very low |
|  | Xu C2016(33) | MD:5.62(4.49, 6.75) | <0.00001 | 0 | serious^a^ | not serious | not serious | serious^b^ | none | Low |
|  | Jiang HL2020(20) | MD:6.18(3.15, 9.21) | <0.0001 | - | serious^a^ | not serious | not serious | serious^b^ | publication bias strongly suspected^d^ | Very low |
|  | Yang XY2018(30) | MD:4.16(0.77, 9.08) | 0.1 | 96 | serious^a^ | serious^c^ | not serious | serious^b^ | none | Very low |
|  | Yang XY2018(30) | MD:4.38(0.53, 9.3) | 0.08 | 96 | serious^a^ | serious^c^ | not serious | serious^b^ | none | Very low |
|  | Chen Y2017(31) | SMD:1.07(0.79, 1.35) | <0.00001 | 0 | serious^a^ | not serious | not serious | serious^b^ | publication bias strongly suspected^d^ | Very low |
| MNCV in tibial nerve | Zhu R2017(32) | MD:1.41(0.33, 3.14) | 0.11 | 0 | serious^a^ | not serious | not serious | serious^b^ | none | Low |
|  | Zhu R2017(32) | MD:1.21(1.07, 3.48) | 0.3 | 53 | serious^a^ | serious^c^ | not serious | serious^b^ | none | Very low |
|  | Xu C2016(33) | MD:6.02(3.87, 8.16) | <0.00001 | 75 | serious^a^ | serious^c^ | not serious | serious^b^ | none | Very low |
|  | Jiang HL2020(20) | MD:7.8(5.4, 10.2) | <0.00001 | - | serious^a^ | not serious | not serious | serious^b^ | publication bias strongly suspected^d^ | Very low |
|  | Yang XY2018(30) | MD:3.8(0.47, 8.08) | 0.08 | 94 | serious^a^ | serious^c^ | not serious | serious^b^ | none | Very low |
|  | Yang XY2018(30) | MD:3.8(0.48, 7.13) | 0.02 | 90 | serious^a^ | serious^c^ | not serious | serious^b^ | publication bias strongly suspected^d^ | Very low |
|  | Chen Y2017(31) | SMD:1.73(1.42, 2.03) | <0.00001 | 0 | serious^a^ | not serious | not serious | serious^b^ | publication bias strongly suspected^d^ | Very low |
| SNCV in peroneal nerve | Huang L2020(25) | SMD:2.28(0.61, 3.96) | <0.00001 | 96 | serious^a^ | serious^c^ | not serious | serious^b^ | none | Very low |
|  | Jiang HL2020(20) | MD:4.29(2.65, 5.93) | <0.00001 | 58 | serious^a^ | serious^c^ | not serious | serious^b^ | none | Very low |
| MNCV in peroneal nerve | Huang L2020(25) | SMD:1.76(0.17, 3.35) | <0.00001 | 87 | serious^a^ | serious^c^ | not serious | serious^b^ | none | Very low |
|  | Jiang HL2020(20) | MD:5.8(4.22, 7.37) | <0.00001 | 59 | serious^a^ | serious^c^ | not serious | serious^b^ | none | Very low |
| MNCV in superficial peroneal nerve | Zhu R2017(32) | MD:2.49(1.69, 3.3) | <0.00001 | 0 | serious^a^ | not serious | not serious | serious^b^ | publication bias strongly suspected^d^ | Very low |
| SNCV in sural nerve | Zhu R2017(32) | MD:2.38(1.19, 3.57) | <0.0001 | 0 | serious^a^ | not serious | not serious | serious^b^ | publication bias strongly suspected^d^ | Very low |
| TCSS | Gao J2021(24) | MD:0.77(1.2, 0.35) | 0.0004 | 26 | serious^a^ | not serious | not serious | serious^b^ | none | Low |
|  | Yang C2020(26) | MD:0.96(1.38, 0.54) | <0.00001 | 0 | serious^a^ | not serious | not serious | serious^b^ | none | Low |
|  | He JB2019(27) | MD:2.07(2.27, 1.86) | <0.0001 | 0 | serious^a^ | not serious | not serious | serious^b^ | none | Low |
|  | Chen Y2017(31) | SMD:1.03(1.36, 0.7) | <0.00001 | 0 | serious^a^ | not serious | not serious | serious^b^ | publication bias strongly suspected^d^ | Very low |
|  | Jiang HL2020(20) | MD:2.01(2.71, 1.3) | <0.00001 | 0 | serious^a^ | not serious | not serious | serious^b^ | none | Low |
|  | Zhou L2023(19) | MD:1.47(1.83, 1.12) | <0.00001 | 51 | serious^a^ | serious^c^ | not serious | serious^b^ | none | Very low |
| VAS | Zhou L2023(19) | MD:2.38(2.76, 2.01) | <0.00001 | 61 | serious^a^ | serious^c^ | not serious | serious^b^ | publication bias strongly suspected^d^ | Very low |
|  | Zhou L2023(19) | MD:1.31(1.6, 1.02) | <0.00001 | 82 | serious^a^ | serious^c^ | not serious | not serious | none | Low |

Explanations:

a. The RCTs included in the MAs had poor methodological quality, inducing a risk of bias.

b. The sample size of continuous variables was <400.

c. The heterogeneity test obtained I^2^＞50%, P<0.01.

d. A small sample size with positive results.

e. The sample size of binary variables was <300.
